# Supplementary material for: Altered Gastric Microbiota and Inflammatory Cytokine Responses in Patients with Helicobacter pylori-Negative Gastric Cancer
Source: Nutrients. 2022 Nov 23;14(23):4981. doi: 10.3390/nu14234981 (PMC9740132; doi:10.3390/nu14234981)

Supplementary Material

Altered Gastric Microbiota and Inflammatory Cytokine Responses in Patients with *Helicobacter pylori*-Negative Gastric Cancer

Han-Na Kim [1,2,†], Min-Jeong Kim [1,†], Jonathan P. Jacobs [3,4] and Hyo-Joon Yang [1,5,*]

1 Medical Research Institute, Kangbuk Samsung Hospital, Sungkyunkwan University School of Medicine, Seoul 03181, Korea.
2 Department of Clinical Research Design and Evaluation, SAIHST, Sungkyunkwan University, Seoul 06355, Korea.
3 Vatche and Tamar Manoukian Division of Digestive Diseases, Department of Medicine, David Geffen School of Medicine at UCLA, Los Angeles, California 90095, United States.
4 Division of Gastroenterology, Hepatology and Parenteral Nutrition, Veterans Administration Greater Los Angeles Healthcare System, Los Angeles, California 90073, United States.
5 Division of Gastroenterology, Department of Internal Medicine and Gastrointestinal Cancer Center, Kangbuk Samsung Hospital, Sungkyunkwan University School of Medicine, Seoul 03181, Korea.
* Correspondence: hyojoonyang@gmail.com
† Han-Na Kim and Min-Jeong Kim contributed equally to this study.

Table S1. Quantitative reverse transcription-polymerase chain reaction (qRT-PCR) primer sequences.

Table S2. Demographics of the patients included in the correlation analysis.

Table S3. Relative abundance of differentially abundant taxa in the patients included in the correlation analysis.

Figure S1. Rarefaction curves based on amplicon sequence variant richness.

Figure S2. Relative percentage abundance of gastric microbiota at the phylum level in the control and cancer groups.

Figure S3. Plot of the out-of-bag (OOB) errors in the random forest classification of the control and cancer groups.

Figure S4. Box plots showing the relative abundance of taxa that differed significantly between the control and cancer groups in Table 2.

Figure S5. Relative expression levels of *IL1B* mRNA in the gastric mucosa of patients with cancer compared to those in the controls among the patients included in the correlation analysis.

Figure S6. Correlation between the altered gastric microbiota and cytokine responses.

Table S1. Quantitative reverse transcription-polymerase chain reaction (RT-qPCR) primer sequences.

Gene	Nucleotide sequence	Accession no.
TNF	F-GCCCAGGCAGTCAGATCATC	NM_000594
	R-AGCTGCCCCTCAGCTTGA	
IL1B	F-GCTGCTCTGGGATTCTCTTCAG	NM_000576
	R-TGGCGAGCTCAGGTACTTCTG	
IL6	F-GGTACATCCTCGACGGCATCT	NM_000600
	R-GTGCCTCTTTGCTGCTTTCAC	
CXCL8	F-CCAGGAAGAAACCACCGGA	NM_000584
	R-GAAATCAGGAAGGCTGCCAAG	
IL10	F-GGGAGAACCTGAAGACCCTCA	NM_000572
	R-TGCTCTTGTTTTCACAGGGAAG	
IL17A	F-CTCATTGGTGTCACTGCTACTG	NM_002190
	R-CCTGGATTTCGTGGGATTGTG	
TGFB1	F-CTGCTGAGGCTCAAGTTAAAAGTG	NM_000660
	R-TGAGGTATCGCCAGGAATTGT	

Table S2. Demographics of the patients included in the correlation analysis.

	Gastric cancer (*n* = 18)	Control (*n* = 29)	*p* value
Age, years, mean ± SD	64.6 ± 8.0	49.2 ± 12.9	< 0.001
Sex, *n* (%)			0.092
Female	6 (33.3)	17 (58.6)	
Male	12 (66.7)	12 (41.4)	
Body mass index, *n* (%)			0.014
< 25 kg/m^2	8 (44.4)	23 (79.3)	
≥ 25 kg/m^2	10 (55.6)	6 (20.7)	
Gastric mucosal atrophy, *n* (%)			< 0.001
Absent	0 (0.0)	20 (69.0)	
Present	18 (100.0)	9 (31.0)	
Intestinal metaplasia, *n* (%)			< 0.001
Absent	2 (11.1)	21 (72.4)	
Present	16 (88.9)	8 (27.6)	

Student's *t*-test and chi-square test were used to compare the two groups. SD, standard deviation.

Table S3. Relative abundance of differentially abundant taxa in the patients included in the correlation analysis**.**

Taxonomic assignment[a]	The overall patients		The patients included in the correlation analysis			
	Relative abundance (mean%)		Relative abundance (mean%)		MaAsLin2 (unadjusted)	MaAsLin2 (adjusted)[b]
	Control	Cancer	Control	Cancer	log_2FC[c]	log_2FC[c]
p_Proteobacteria;						
o_Gammaproteobacteria;						
c_Pasteurellales	6.29%	4.45%	6.13%	5.19%	−0.828	−1.101
f_Pasteurellaceae	6.29%	4.45%	6.13%	5.19%	−0.828	−1.101
g_*Haemophilus*	6.29%	4.45%	6.13%	5.19%	−0.828	−1.101
s_parainfluenzae	4.99%	2.92%	4.86%	3.41%	−0.801	−0.634
p_Proteobacteria;						
o_Epsilonproteobacteria;						
c_Campylobacterales;						
f_Campylobacteraceae;						
g_*Campylobacter*	1.04%	0.38%	1.01%	0.44%	−2.364	−1.973
p_Firmicutes;						
o_Bacilli;						
c_Lactobacillales;						
f_Lactobacillaceae;						
g_*Lacticaseibacillus;*	0.29%	1.24%	0.45%	1.35%	0.607	0.416
s_*casei*	0.29%	1.24%	0.45%	1.35%	0.607	0.416

[a]NCBI-RefSeq database was used for taxonomic assignment. [b]Adjusted for age, sex, and body mass index. [c]Coefficients for log-transformed relative abundance of each taxon in linear models using MaAsLin2. The control group was set as the reference group and compared to the cancer group. MsAsLin, multivariate association with linear models; FC, fold change.

Figure S1. Rarefaction curves based on amplicon sequence variant richness. Number of observed features indicates that 6,814 sequences per sample are sufficient for capturing the α-diversity of microbial communities in the control and cancer groups. X-axis shows the number of sequences per sample.

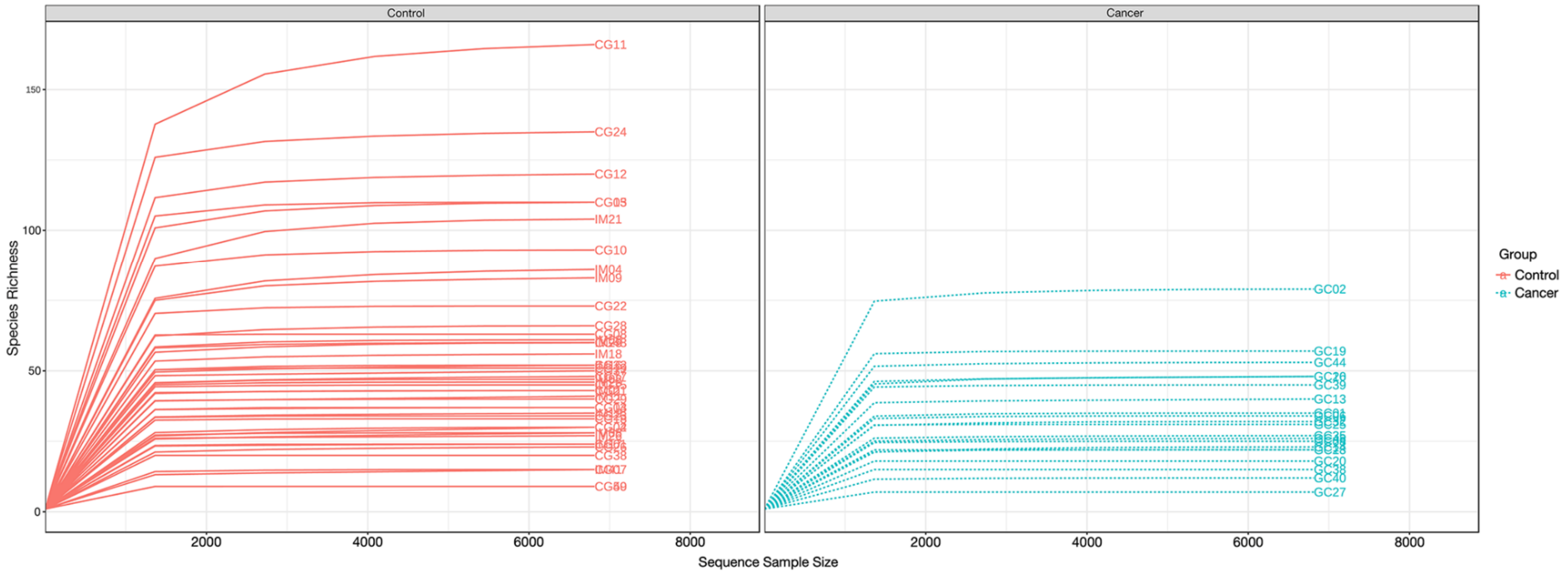

Figure S2. Relative percentage abundance of gastric microbiota at the phylum level in the control and cancer groups.

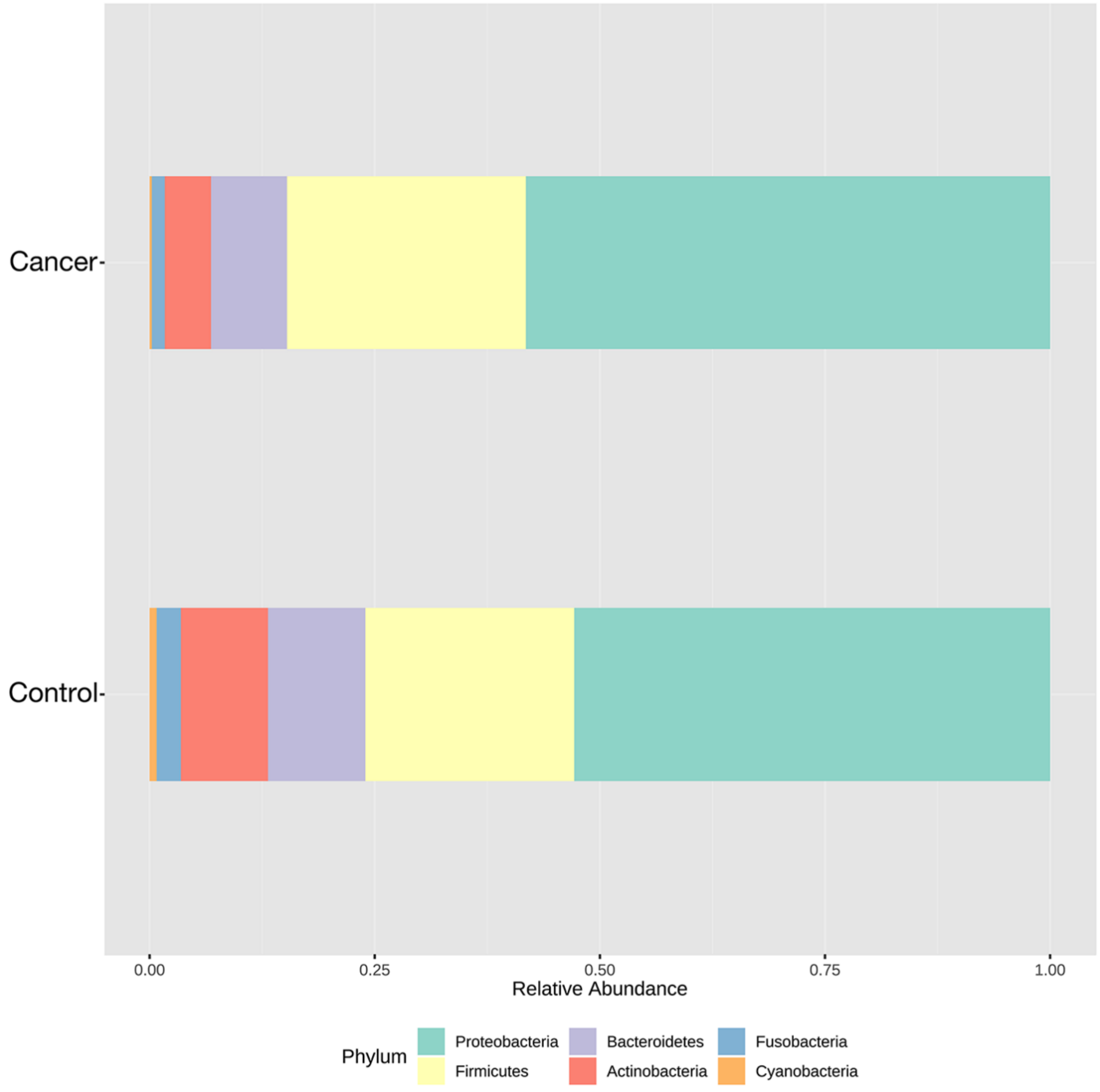

Figure S3. Plot of the out-of-bag (OOB) errors in the random forest classification of the control and cancer groups. Red, green, and blue lines represent all samples, control group, and cancer group, respectively. The classification performance for the control and cancer groups is shown in the table to the right.

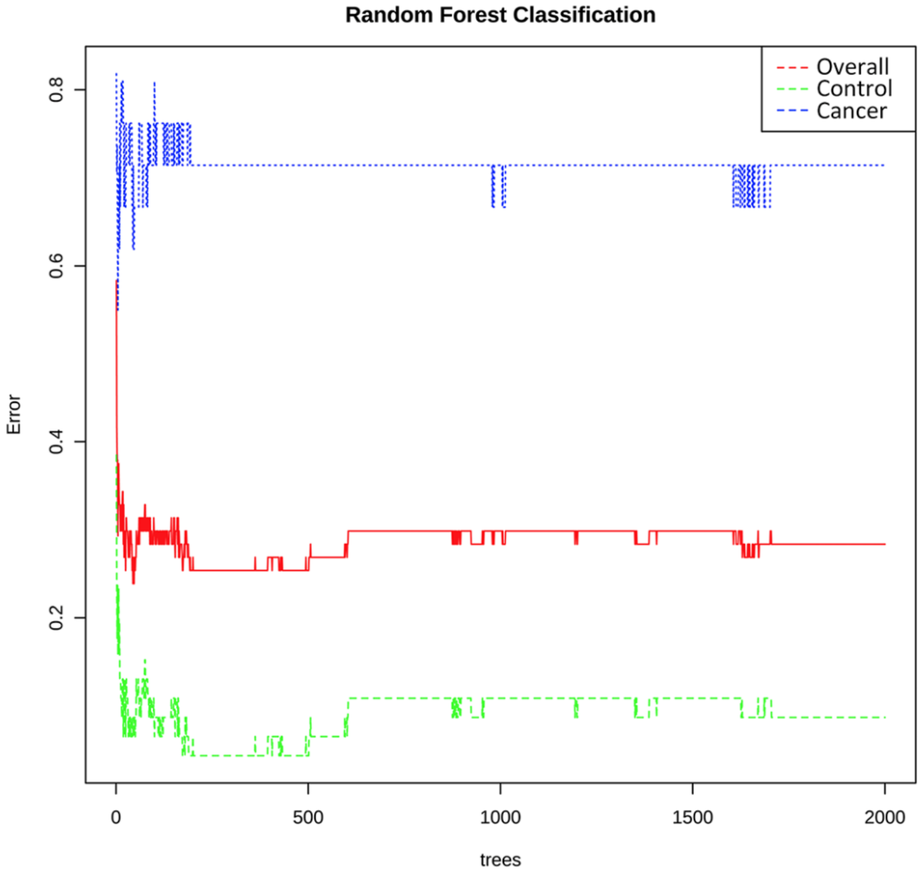

The OOB error is 0.254

	Control	Case	class.error
Control	43	3	0.0652
Cancer	14	7	0.667

Figure S4. Box plots showing the relative abundance of taxa that differed significantly between the control and cancer groups in Table 2. Taxa detected to be significantly different relative to more than 70% of other taxa at each taxon level (detected > 0.7) in the analysis of comparison of microbiomes (ANCOM)-2 analysis (false discovery rate [FDR] < 0.1).

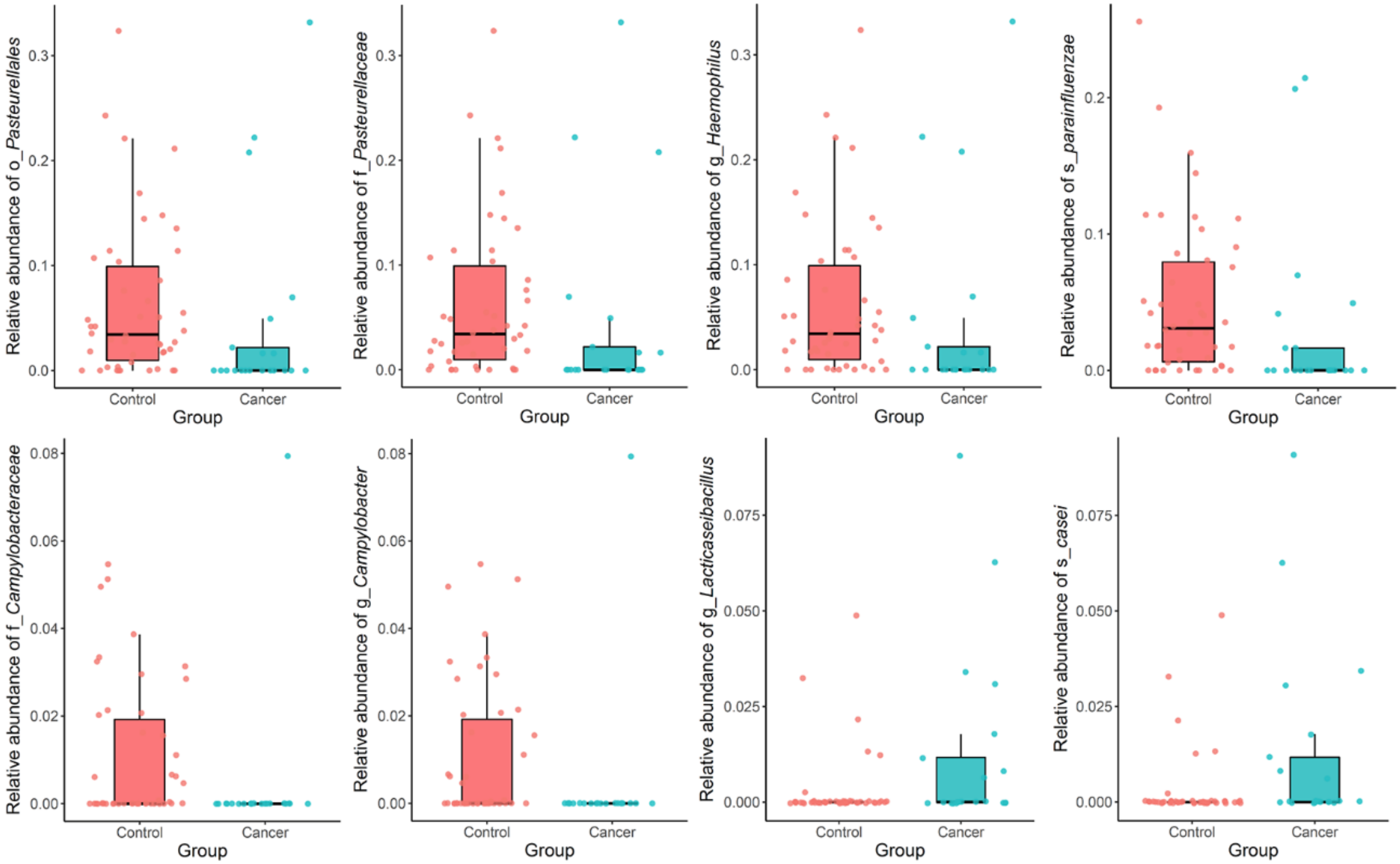

Figure S5. Relative expression levels of *IL1B* mRNA in the gastric mucosa of patients with cancer compared to those in the controls among the patients included in the correlation analysis. Levels of relative mRNA expression of *TNF, IL1B, IL6, CXCL8, IL10, IL17A,* and *TGFB1* were compared (**A**) between the control and cancer groups and (**B**) between patients with chronic gastritis (CG), intestinal metaplasia (IM), and gastric cancer (GC). Levels were presented as fold change compared to the reference group (control and CG, respectively) and standard errors. *$p < 0.05$.

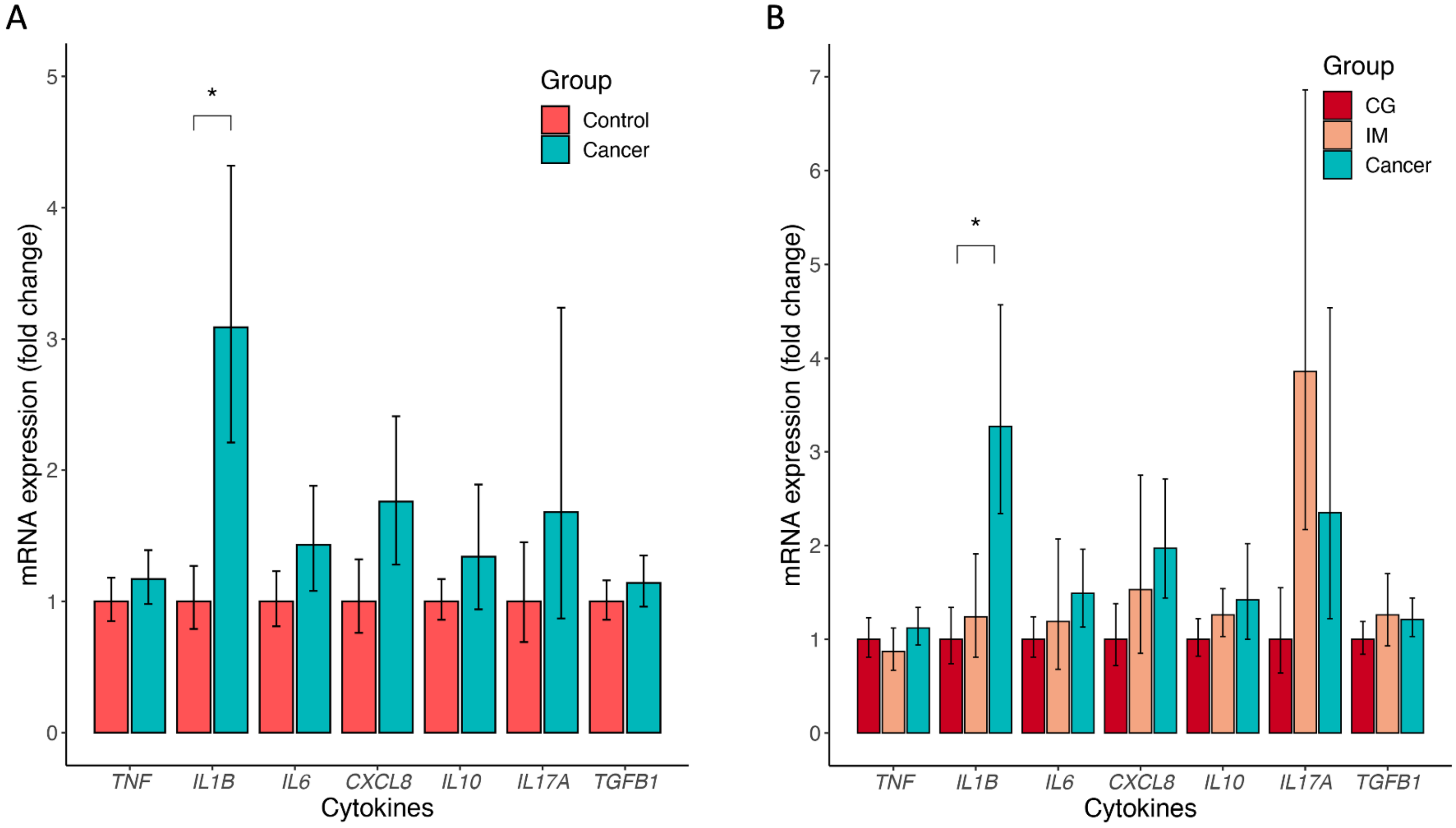

Figure S6. Correlation between the altered gastric microbiota and cytokine responses. The differentially associated taxa between GC and control groups, (A) *Campylobacter*, (B) *Haemophilus*, and (C) *Lacticaseibacillus*, and the relative mRNA expression of *IL1B* were evaluated.

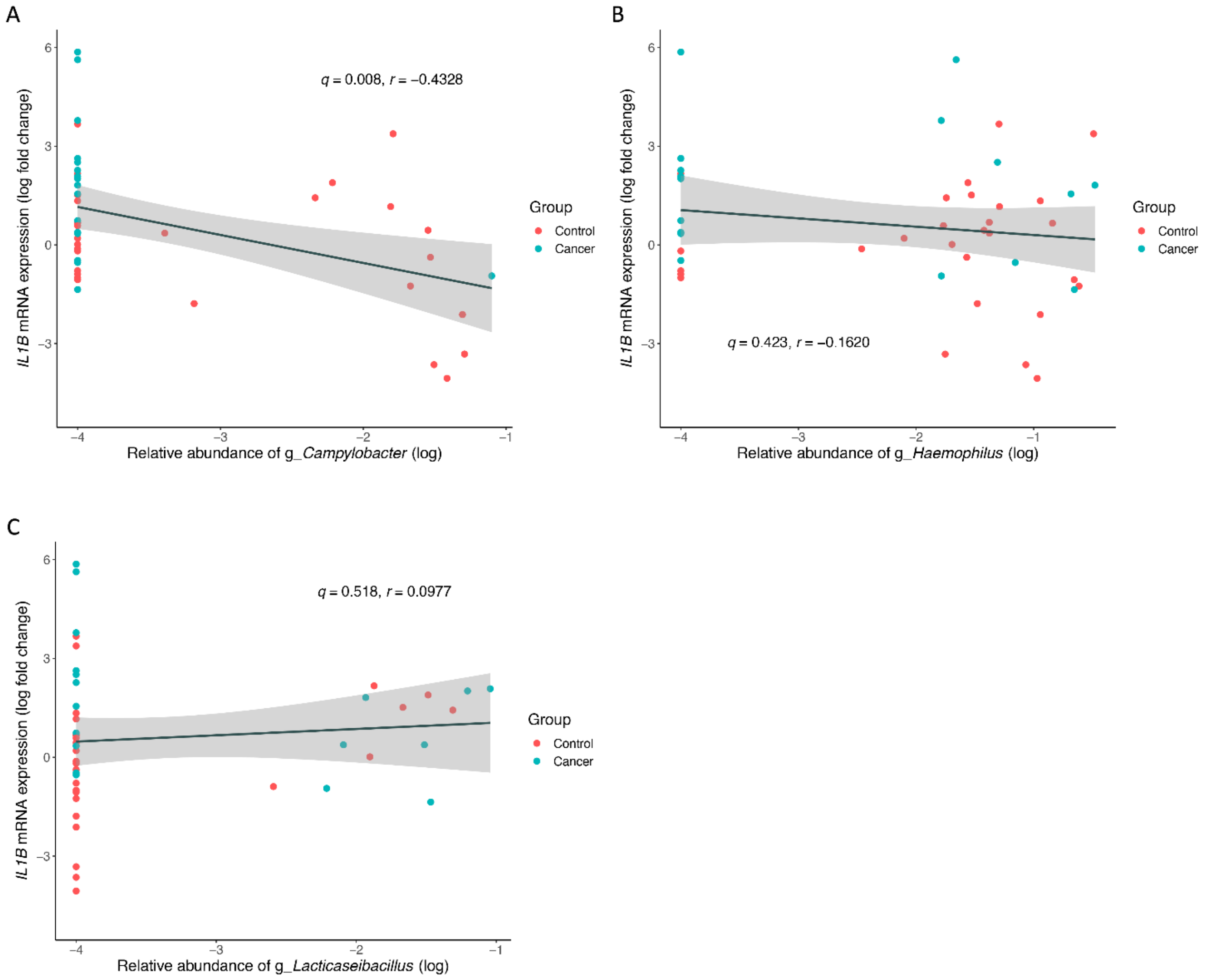

Supplement: Supplementary file 1 [file nutrients-14-04981-s001.zip › nutrients-2030628-supplementary.pdf]
